# Systemic and Pulmonary Vascular Remodelling in Chronic Obstructive Pulmonary Disease

**DOI:** 10.1371/journal.pone.0152987

**Published:** 2016-04-05

**Authors:** Mariana Muñoz-Esquerre, Marta López-Sánchez, Ignacio Escobar, Daniel Huertas, Rosa Penín, María Molina-Molina, Frederic Manresa, Jordi Dorca, Salud Santos

**Affiliations:** 1 Department of Pulmonary Medicine, Bellvitge University Hospital -IDIBELL, University of Barcelona, L’Hospitalet de Llobregat, Barcelona, Spain; 2 Department of Thoracic Surgery, Bellvitge University Hospital -IDIBELL, University of Barcelona, L’Hospitalet de Llobregat, Barcelona, Spain; 3 Department of Pathology, Bellvitge University Hospital -IDIBELL, University of Barcelona, L’Hospitalet de Llobregat, Barcelona, Spain; 4 Biomedical Research Networking Center Consortium -Respiratory Diseases (CIBERES), Barcelona, Spain; Research Center Borstel, GERMANY

## Abstract

**Background:**

Chronic Obstructive Pulmonary Disease (COPD) is associated with subclinical systemic atherosclerosis and pulmonary vascular remodelling characterized by intimal hyperplasia and luminal narrowing. We aimed to determine differences in the intimal thickening of systemic and pulmonary arteries in COPD subjects and smokers. Secondary aims include comparisons with a non-smokers group; determining the clinical variables associated with systemic and pulmonary intimal thickening, and the correlations between systemic and pulmonary remodelling changes.

**Methods:**

All consecutive subjects undergoing lung resection were included and divided into 3 groups: 1) COPD, 2) smokers, and 3) non-smokers. Sections of the 5th intercostal artery and muscular pulmonary arteries were measured by histo-morphometry. Four parameters of intimal thickening were evaluated: 1) percentage of intimal area (%IA), 2) percentage of luminal narrowing, 3) intimal thickness index, and 4) intima-to-media ratio.

**Results:**

In the adjusted analysis, the systemic arteries of COPD subjects showed greater intimal thickening (%IA) than those of smokers (15.6±1.5% vs. 14.2±1.6%, p = 0.038). In the pulmonary arteries, significant differences were observed for %IA between the 2 groups (37.3±2.2% vs. 29.3±2.3%, p = 0.016). Among clinical factors, metabolic syndrome, gender and COPD status were associated with the systemic intimal thickening, while only COPD status was associated with pulmonary intimal thickening. A correlation between the %IA of the systemic and pulmonary arteries was observed (Spearman’s rho = 0.46, p = 0.008).

**Conclusions:**

Greater intimal thickening in systemic and pulmonary arteries is observed in COPD patients than in smokers. There is a correlation between systemic and pulmonary vascular remodelling in the overall population.

## Introduction

The most important comorbidity associated with chronic obstructive pulmonary disease (COPD), due to its impact on prognosis and mortality, is cardiovascular disease (CVD).[1–2] In this setting, previous studies suggest that COPD subjects have an increased risk of ischemic heart disease independent of smoking, age or gender.[3–5] However, the underlying mechanisms of this frequent association (between COPD and CVD) have not been completely elucidated.[4] Although the pathogenesis of atherosclerosis is complex, low-grade systemic inflammation, which is present in COPD and in CVD, could be one of the centrepiece events leading to systemic vascular remodelling and plaque formation.[3] Moreover, the remodelling of pulmonary vessels is a well-recognized finding in COPD.[6–7] This process is characterized by the migration and proliferation of vascular smooth muscle cells, inducing intimal hyperplasia and, therefore, subsequent luminal narrowing.[7] These pulmonary changes are mainly caused by sustained inflammatory process triggered by smoke exposure.[8]

However, to date, to the best of our knowledge, no previous histological study has evaluated vascular remodelling changes, such as the intimal thickening of the systemic arteries of COPD subjects, and its relationship with the presence of pulmonary intimal hyperplasia. The principal aim of study was to determine differences in intimal thickening in terms of the percentage of intimal area (%IA) of systemic arteries in COPD subjects and smokers. Secondary aims include an evaluation of differences in other intimal thickening parameters, all parameter comparisons with a non-smoker group, the determination of the variables associated with systemic and pulmonary intimal thickening and an evaluation of the correlation between systemic and pulmonary changes in the overall population.

## Methods

### Population

This was a prospective investigation, conducted in consecutive subjects who required lung resection for the treatment of lung cancer recruited from the Department of Pulmonary Medicine of University Hospital of Bellvitge (L’Hospitalet de Llobregat, Spain). Demographic and clinical data were obtained from patients’ medical records. A preoperative pulmonary function test was performed in all subjects. Patients were divided into three groups according to their smoking history and pulmonary function tests: 1) COPD subjects (all of whom were current or former smokers with airflow limitation), 2) smokers (current or former smokers with normal lung function), and 3) non-smokers (never-smokers). Exclusion criteria were the presence of any pulmonary disease other than COPD and previous treatment with chemotherapy or radiotherapy regimens or previous lung surgery. The definition of COPD was established following the current guidelines.[1] The study was approved by the local ethics committee “Comitè Ètic d’ Investigació Clínica del Hospital de Bellvitge, N° PR006/11”, and performed in accordance with the Declaration of Helsinki. All patients signed an informed consent form.

### Sample collection

To evaluate systemic circulation, sections of the 5th posterior intercostal (IC) artery (1–1.5cm in length) were taken during the thoracotomy incision. To assess pulmonary circulation, lung samples were obtained from the piece of lung resection, as far away as possible from the tumour, and muscular pulmonary arteries with an external diameter between 100 to 500 μm were considered in the analyses. Both tissues (IC artery and lung samples) were fixed overnight in 4% paraformaldehyde following established methods of fixation and preparation of samples for morphometry.[9] Venous blood samples were collected from all subjects. Fasting blood glucose (FBG), total cholesterol, triglycerides, high-density lipoprotein cholesterol (cHDL), low-density lipoprotein cholesterol (cLDL), and blood cell counts were determined with standard laboratory methods. Metabolic syndrome (MetS) was defined as the presence of three or more of the metabolic parameters in accordance with current criteria as detailed in S1 File.[10]

### Morphometric analysis

**Fig 1:**
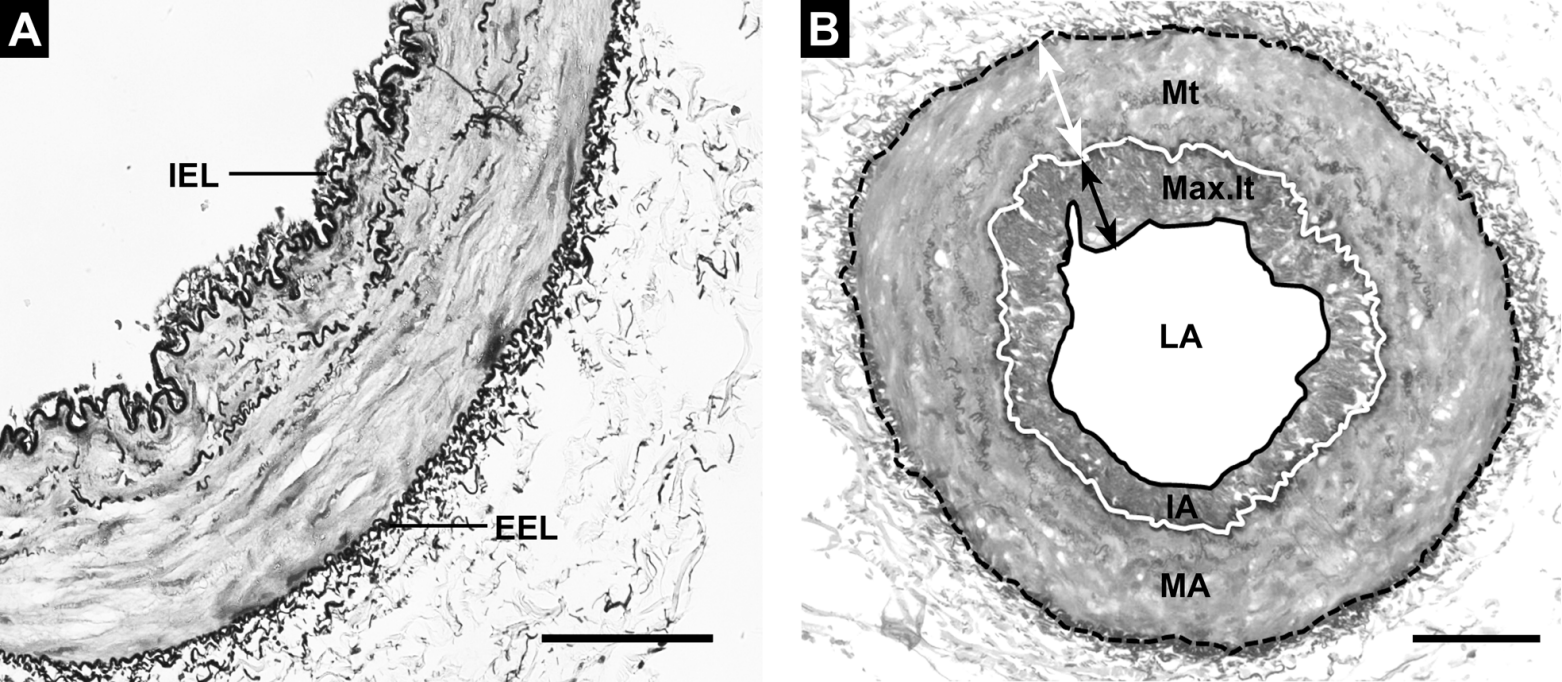
Histologic and morphometric analyses. **A.** Fifth intercostal artery showing almost no intimal thickness and muscular media layer. Note also the absence of elastic laminas except for the internal elastic lamina (IEL) and external elastic lamina (EEL). **B**. Methods used for morphometric analyses as described in the text. A solid white line represents the IEL and the discontinuous black line shows the EEL. The area enclosed by the solid black line is the lumen area (LA), the area enclosed by the solid white line is the combined lumen + intima area (IA) and the area enclosed by the discontinuous black line is the lumen + intima + media area (MA). A double-headed black arrow represents maximal intimal thickness (Max.It). The double-headed white arrow shows the medial thickening (Mt) at maximal intimal thickness. Elastin-orcein stain, scale bar = 100μm.

Tissue sections were stained with elastin-orcein stain to localize elastin fibres and to differentiate the external elastic lamina (EEL) and internal elastic lamina (IEL) from all arteries (Fig 1A). Only vessels with complete elastic laminas were evaluated. Using a digital micro-imaging device (Leica DMD108, Leica Microsystems GmbH- Germany), EEL and IEL and the inner aspect of the intimae were outlined as previously described [9] by two different observers working blindly with regard to the study groups. In addition, artery diameters and areas of lumen, IEL, intimal and medial layer were calculated (Fig 1B). In order to evaluate the effect of vascular contraction and tissue shrinkage during manipulation or fixation, an index of narrowing was estimated for each artery (S1 File).[11] In line with previous studies of vascular remodelling and atherosclerosis and following stereological methods for vascular evaluation,[9, 12–13] four methods were used to analyze the degree of intimal thickening: 1) percentage of intimal area (%IA = 100Xintimal area/measured total area or area encompassed by the EEL), 2) percentage of luminal narrowing (%LN = 100Xintimal area/IEL area) which consider IEL as the surface reference, 3) intimal thickness index (ITI = intimal area/medial area), and 4) intima-to-media ratio (IMR = width of intima at maximal intimal thickness/width of media at maximal intimal thickness).

### Study endpoints and sample size calculation

The primary endpoint of this study was the difference in the %IA of systemic arteries in COPD patients compared to smokers. Assuming a standard deviation of 5(%), a sample size of 12 subjects per group was needed to detect a minimal difference between groups of 6%; with 80% power and a two-tailed p-value less than 0.05. Considering an approximate 20% dropout rate (e.g. inadequate samples for morphometric measurements), the inclusion of 15 subjects by group was allowed to ensure that data from 12 patients was available for analysis. Secondary endpoints included: 1) between-group comparisons for %LN, ITI and IMR of systemic arteries, 2) all parameter comparisons with a non-smoker group, 3) associations between clinical variables and %IA of systemic and pulmonary arteries, and 4) correlations between systemic and pulmonary remodelling parameters in the overall population.

### Statistical methods

For baseline characteristics, continuous variables were expressed as mean±SD or by median and interquartile range, whether a normal distribution was assumed or not (Kolmogorov-Smirnov test), respectively. Comparisons of continuous variables were performed with the Student’s t-test or Mann-Whitney’s U test as appropriate, while qualitative variables were compared with the chi-square test or Fisher’s exact test (if the expected cell frequencies were lower than 5). An ANOVA method with a general linear model (GLM) was used to evaluate overall comparisons. The primary endpoint (difference in the %IA of systemic arteries in COPD patients compared to smokers), and all other between-groups comparisons were performed using the least significant difference method with GLM. All adjusted analyses were performed with an ANCOVA method by GLM, using as covariates those variables associated with %IA in the overall population of the study (p<0.10): gender, MetS, cHDL and circulating leukocytes. Results are reported as least squares means (LSM) ± standard error of the mean (SEM) for the analyses detailed above. The linear regression analyses performed to determine which clinical factors were associated with the intimal thickening of systemic and pulmonary vessels are detailed in S1 File. Spearman’s correlation coefficients (p) were used to assess the relationships between pairs of continuous intimal thickening parameters in systemic and pulmonary arteries. A two-tailed p-value of <0.05 was considered as statistically significant in all the tests performed. Statistical analysis was performed using PASW Statistics v18.0 software (SPSS Inc., Chicago, IL).

## Results

Consecutive samples from 48 patients undergoing lung resection surgery were prospectively included in this protocol. However, six of them (12.5%) were excluded due to poor conservation of vessel architecture (n = 4) or incomplete measurable elastic laminas despite several attempts at the reorientation of the sample (n = 2).Therefore, 42 subjects with valid IC arteries were analysed. Seventeen in the group of COPD (current = 11 and former smokers = 6), 14 in the smokers group (current = 5 and former smokers = 9), and 11 in the non-smokers group. Overall, baseline variables were mostly well balanced between groups (data is summarized in Table 1). However, some differences were observed in the COPD group compared to smokers, where higher tobacco consumption (pack-years) was observed in COPD group, and a higher prevalence of metabolic syndrome and diabetes mellitus was seen in smokers. In the comparisons performed with the non-smokers group, predominance of male gender and the presence of aortic calcifications in the COPD and smokers groups were observed.

**Table 1:** Baseline demographics by groups.

Parameters	COPD (N = 17)	Smokers (N = 14)	Non smokers (N = 11)	Overall p-value
Male gender, n (%)	16 (94.1)^†^	13 (92.9)	4 (36.4)	<0.001
Age, years	63.4 [57.7–68.8]	58.3 [50.0–65.9]	66.1 [49.5–68.9]	0.524
BMI, kg/m^2^	24.0 [21.7–27.7]	27.1 [25.0–30.4]	28.9 [21.5–30.1]	0.187
Pack-years	45 [39–60]^†^^,^^‡^	37.5 [20–41.3]	0 [0–0]	<0.001
Current smoking, n (%)	11 (64.7)^†^	5 (35.7)	0	0.003
Emphysema in CT, n (%)	12 (70.6)^†^^,^^‡^	4 (28.6)	0	0.001
Aortic calcifications, n (%)	13 (76.5)^†^	8 (57.1)	3 (27.3)	0.037
Systemic hypertension, n (%)	5 (29.4)	7 (50.0)	4 (36.4)	0.497
Diabetes Mellitus, n (%)	1 (5.9)^‡^	7 (50)	0	0.001
Renal failure, n (%)	0	0	0	-
Met.S, n (%)	6 (35.3)^‡^	11 (78.6)	3 (27.3)	0.016
Lipid lowering, n (%)	5 (29.4)	5 (35.7)	3 (27.3)	0.888
ACEIs/ARBs, n (%)	5 (29.4)	8 (57.1)	3 (27.3)	0.198
FEV_1_ Post-BD, % predicted	62.0 [53.9–82.0]^†^^,^^‡^	97.7 [80.0–103.5]	107.0 [89.3–119.8]	<0.001
FEV_1_/FVC Post-BD, %	54.9 [43.3–67.6]^†^^,^^‡^	76.3 [72.6–81.2]	77.2 [73.7–81.5]	<0.001
D_LCO_, % predicted	68.3 [53.0–76.5]^†^^,^^‡^	87.1 [71.5–102.3]	84.4 [72.1–103]	0.001
LABA, n (%)	6 (35.3)^‡^	-	-	0.006
Inhaled CS, n (%)	5 (29.4)^‡^	-	-	0.015
WC, cm	83.1 [76.3–93.8]	90.9 [86.5–95.7]	88.4 [78.4–99.1]	0.299
SBP, mmHg	130 [120–137]	130 [121–138]	126 [120–135]	0.835
DBP, mmHg	73 [70–82]	74 [70–79]	70 [60–78]	0.340
cHDL, mmol/L	1.23 [0.90–1.37]^†^	1.19 [0.78–1.37]	1.55 [1.18–2.07]	0.030
cLDL, mmol/L	2.51 [1.91–3.19]	2.26 [1.23–3.05]	2.96 [2.24–3.50]	0.187
Total cholesterol, mmol/L	4.41 [3.68–5.03]	4.44 [3.70–4.99]	5.55 [3.88–5.86]	0.516
Triglycerides, mmol/L	1.25 [0.83–1.86]	1.99 [1.61–2.39]	1.60 [1.17–1.88]	0.131
FBG, mmol/L	5.6 [4.8–6.6]	6.7 [5.2–7.5]	5.4 [4.9–6.5]	0.074
Leukocytes count, x10E9/L	8.2 [7.7–9.9]^†^	7.8 [6.8–8.8]	6.3 [5.7–8.3]	0.045

Data are presented as median [25^th^-75^th^ percentile]. BMI: body mass index, Met.S: Metabolic syndrome, ACEIs/ARBs: angiotensin-converting enzyme inhibitors or angiotensin II receptor blockers, FEV_1_: forced expiratory volume in one second, BD: bronchodilator, FVC: forced vital capacity, D_LCO_: diffusing capacity of the lungs for carbon monoxide, LABA: long acting β-agonists, CS: inhaled corticosteroids, WC: waist circumference; SBP: systolic blood pressure, DBP: diastolic blood pressure, cHDL: high-density lipoprotein cholesterol, cLDL: low-density lipoprotein cholesterol, FBG: fasting blood glucose.

^†^ means a p-value <0.05 for the difference between COPD and non-smokers group, and

^‡^ means a p-value <0.05 for the difference between COPD and smokers group.

### Morphometric measurements and severity indices of intimal thickening

**Fig 2:**
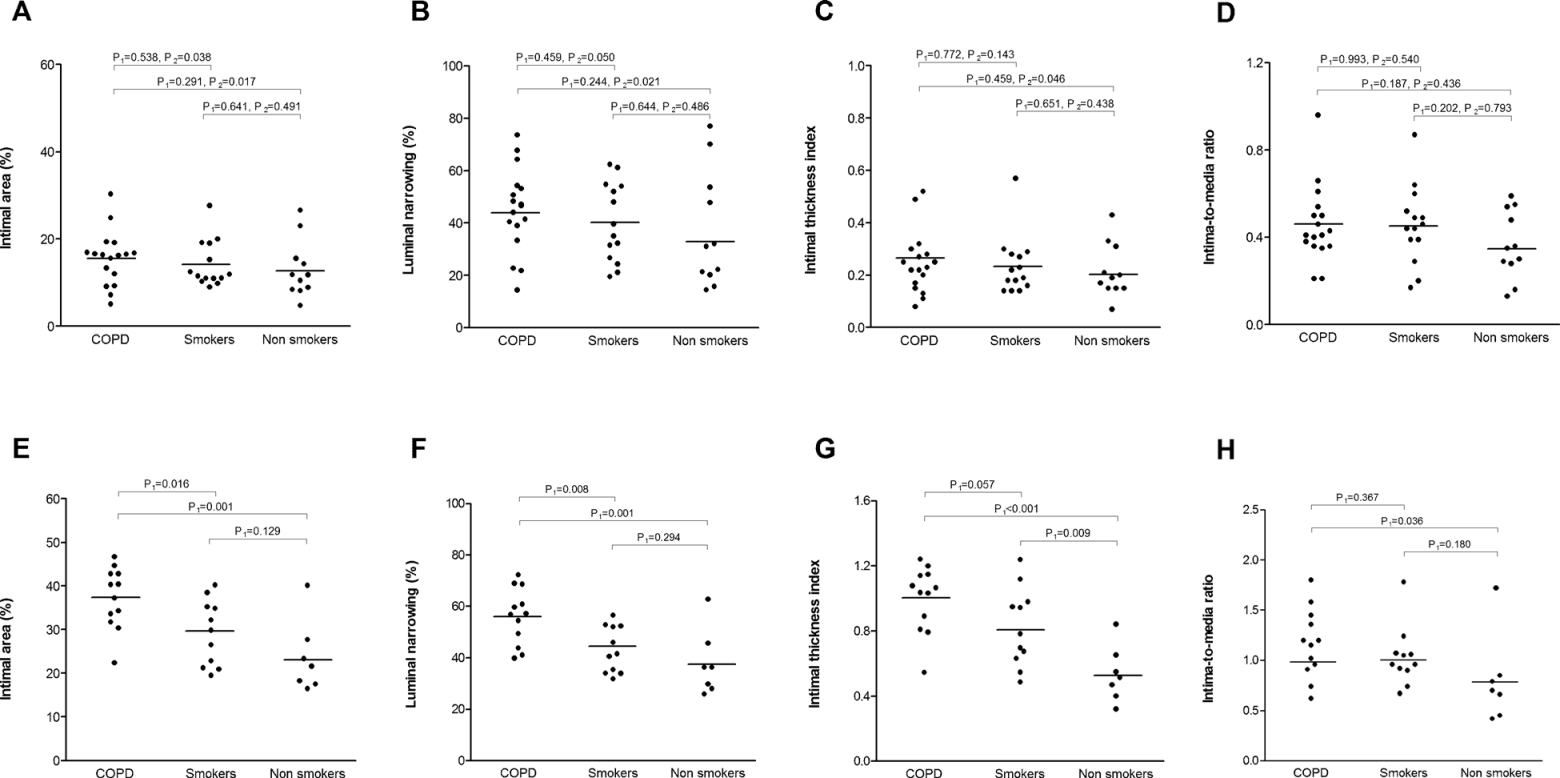
Severity indices of intimal thickening in systemic and pulmonary vessels, by groups. **A-D** represents individual data of systemic intimal thickening parameters: intimal area, luminal narrowing, intimal thickness index and intima-to-media ratio, respectively. **E-H** represents individual data of pulmonary intimal thickening parameters: intimal area, luminal narrowing, intimal thickness index and intima-to-media ratio, respectively. Horizontal bars indicate least squares mean values. P values for the pairwise comparisons using the least significant difference method with general linear model. P1: P-value for unadjusted analysis, P2: P-value for adjusted analysis using as covariables: gender, MetS, cHDL and circulating leukocytes.

Morphometric measurements of systemic arteries showed no differences in the dimensions of vessels or areas between groups (Table 2). The severity indices of intimal thickening as evaluated by non-adjusted analyses (%IA, %LN, ITI and IMR) were not significantly different between COPD and smokers. However, in the adjusted analysis %IA was significantly higher in COPD subjects compared to smokers. %LN and ITI showed a numerically increasing trend in the COPD group compared to the smokers group, while not attaining statistical significance. Three of the four indices of intimal thickening (%IA, %LN and ITI) were significantly higher in the COPD group than in the group of non-smokers. Covariates included in the adjusted analyses were gender, MetS, cHDL and circulating leukocytes. Individual data and p-values of unadjusted and adjusted analysis of intimal thickening parameters by groups are represented in Fig 2A–2D. No severe lesions with calcification were observed in the arteries examined.

**Table 2:** Morphometric measurements and severity indices of intimal thickening in systemic and pulmonary arteries by groups.

**Measurements**	**Systemic arteries**			
	**COPD**	**Smokers**	**Non smokers**	**Overallp-value**
Measured external diameter, μm	557 [485–668]	506 [440–599]	505 [427–632]	0.385
Measured total area, mm^-2^x10^-3^	239 [162–337]	199 [143–254]	189 [141–277]	0.338
Index of narrowing^a^	0.27 [0.24–0.33]	0.30 [0.23–0.34]	0.28 [0.27–0.32]	0.845
Lumen area, mm^-2^x10^-3^	49.9 [25.9–69.6]	47.4 [24.5–59.2]	58.8 [20.1–92.2]	0.811
Intima area, mm^-2^x10^-3^	32.0 [19.6–57.0]	26.8 [17.7–37.7]	23.4 [17.5–37.1]	0.327
Muscular area, mm^-2^x10^-3^	157.2 [115.5–201.9]	117.1 [96.4–161.1]	113.3 [72.9–164.8]	0.169
**Severity Indices**^b^				
% intimal area	15.6 ± 1.5^†,^^‡^	14.2 ± 1.6	13.1 ± 1.8	0.022
% luminal narrowing	44.9 ± 4.2^†^	40.2 ± 4.7	36.9 ± 5.3	0.030
Intimal thickness index	0.25 ± 0.03^†^	0.24 ± 0.03	0.21 ± 0.03	0.091
Intima to media ratio	0.46 ± 0.04	0.46 ± 0.05	0.37 ± 0.05	0.678
	**Pulmonary arteries**			
	**COPD**	**Smokers**	**Non smokers**	**Overallp-value**
N° of arteries measured by patient	10.2 ± 1.2	13.2 ± 1.8	8.7 ± 2.3	0.212
Measured external diameter, μm	294.2 [256.2–362.1]	323.9 [303.5–406.4]	327.5 [314.8–360.1]	0.176
Measured total area, mm^-2^x10^-3^	64.0 [49.8–99.1]	77.4 [64.2–121.6]	74.3 [65.8–84.7]	0.237
Index of narrowing^a^	0.30 [0.28–0.32]	0.31 [0.28–0.32]	0.30 [0.29–0.34]	0.862
Lumen area, mm^-2^x10^-3^	18.0 [12.9–23.4]^‡^	27.9 [25.3–37.3]	26.9 [16.9–35.4]	0.013
Intima area, mm^-2^x10^-3^	24.0 [18.2–32.7]	21.2 [16.4–33.3]	15.4 [12.2–21.1]	0.092
Muscular area, mm^-2^x10^-3^	21.8 [17.0–34.9]	27.5 [21.1–45.4]	30.3 [26.5–33.8]	0.291
**Severity Indices**^b^				
% intimal area	37.3 ± 2.2^†,^^‡^	29.3 ± 2.3	23.6 ± 2.8	0.002
% luminal narrowing	56.1 ± 3.1^†,^^‡^	43.4 ± 3.2	37.9 ± 4.1	0.003
Intimal thickness index	0.99 ± 0.06^†^	0.82 ± 0.06	0.54 ± 0.08	<0.001
Intima to media ratio	1.17 ± 0.10^†^	1.03 ± 0.11	0.80 ± 0.13	0.107

Data are presented as median [IQR] or LSM ± SEM. The reported p-value comes from the overall comparison with ANCOVA method with a general linear model using as covariables: gender, MetS, cHDL and circulating leukocytes.

Between-group comparisons were performed using the least significant difference method, where ^†^ means a p-value <0.05 for the difference between COPD and non-smokers group, and

^‡^ means a p-value <0.05 for the difference between COPD and smokers group.

^a^Index of narrowing is estimated as the ratio between the measured total area and that extrapolated from the theoretical distended diameter: [*theoretical diameter = length of the external elastic lamina / pi (π)*].

^b^The severity indices were calculated using the following formulas: % intimal area = 100 X intimal area/measured total area (area encompassed by the external elastic lamina); % luminal narrowing = 100 X intimal area/internal elastic lamina area; Intimal thickness index = intimal area/medial area; and Intima to media ratio = width of intima at maximal intimal thickness/width of media at maximal intimal thickness.

In muscular pulmonary arteries, the morphometric valuation revealed similar vessel dimensions among groups (Table 2). However, it was observed that two of the intimal thickening parameters (%IA and %LN) were significantly higher in the COPD group than in the smokers group. Furthermore, each of the 4 severity indices was significantly superior in the COPD group compared to the non-smokers group. In addition, a greater degree of intimal thickening (ITI) was observed in the smokers group compared to the non-smokers group. It is noteworthy that the only clinical factor associated with the %IA of pulmonary vessels in the regression analysis was COPD status. Therefore, the data reported above are from the unadjusted analysis. Plots of individual data for intimal thickening of pulmonary arteries by groups are represented in Fig 2E–2H. There was agreement in morphometric measurements between the two observers with an intra-class correlation coefficient (ICC) of 0.907 (CI from 0.78 to 0.96, p<0001).

### Factors associated with intimal thickening in systemic and pulmonary arteries

The results of univariate and multivariate regression analyses of the factors associated with intimal thickening in systemic arteries (%IA) are summarized in Table 3. Based on these results, five variables were identified (p<0.10) as possible risk factors: male gender, leukocytes count and cHDL (related to lower %IA) and MetS and COPD (related to higher %IA). In the analyses explained above, the presence of Diabetes Mellitus and the pack-years were not associated to %IA (Table 3). In the multivariate analysis, only MetS, gender and COPD were significantly associated with %IA. Table 4 shows the clinical variables tested as possible factors associated with pulmonary intimal thickening (%IA). Only COPD was significantly associated with %IA.

**Table 3:** Linear regression analyses for associations between clinical variables and the intimal thickening (%IA) of systemic arteries in the overall population.

Dependent variable:% Intimal area	Univariateβ coefficient [95% CI]	p-value	Multivariateβ coefficient [95% CI]	p-value
Male gender	-6.33 [11.20- -1.46]	0.013	-6.64 [-11.14- -2.14]	0.005
Age	0.061 [-0.14–0.26]	0.543		
BMI	0.15 [-0.35–0.64]	0.541		
Pack-years	-0.03 [-0.21–0.16]	0.758		
Emphysema	0.173 [-6.85–7.20]	0.960		
Systemic hypertension	-1.31 [-5.82–3.20]	0.555		
Diabetes Mellitus	0.86 [-6.13–7.84]	0.801		
Metabolic syndrome	5.78 [1.84–9.73]	0.006	5.52 [2.21–8.82]	0.002
Lipid lowering	1.48 [-4.03–6.98]	0.585		
ACEIs/ARBs	-0.20 [-8.65–8.25]	0.961		
cHDL	-3.59 [-7.79–0.60]	0.091	-3.32 [-7.10–0.45]	0.083
cLDL	0.62 [-1.81–3.14]	0.618		
Triglycerides	-1.24 [-4.71–2.24]	0.471		
Leukocytes	-0.89 [-1.94–0.16]	0.095	-0.64 [-1.55–0.27]	0.164
COPD	4.78 [0.53–9.03]	0.029	4.96 [1.43–8.48]	0.007

BMI: body mass index, ACEIs/ARBs: angiotensin-converting enzyme inhibitors or angiotensin II receptor blockers, cHDL: high-density lipoprotein cholesterol, cLDL: low-density lipoprotein cholesterol.

**Table 4:** Linear regression analyses for associations between clinical variables and the intimal thickening (%IA) of pulmonary arteries in the overall population.

Dependent variable:% Intimal area	Univariateβ coefficient [95% CI]	p-value
Male gender	-2.77 [-11.78–6.25]	0.530
Age	0.109 [-0.23–0.45]	0.515
BMI	-0.06 [-1.09–0.97]	0.902
Pack-years	0.09 [-0.04–0.23]	0.163
Emphysema	-1.79 [-9.83–6.24]	0.647
Systemic hypertension	-1.21 [-12.65–10.23]	0.827
Diabetes Mellitus	-4.61 [-12.22–2.99]	0.223
Metabolic syndrome	0.94 [-9.01–10.89]	0.844
Lipid lowering	3.37 [-2.47–9.20]	0.247
ACEIs/ARBs	1.52 [-8.16–11.19]	0.746
Inhaled CS	4.32 [-5.91–14.55]	0.391
Leukocytes	-0.86 [-2.38–0.65]	0.252
COPD	10.23 [4.37–16.09]	0.001

BMI: body mass index, ACEIs/ARBs: angiotensin-converting enzyme inhibitors or angiotensin II receptor blockers, CS: corticosteroids.

### Correlations between systemic and pulmonary intimal thickening

**Fig 3:**
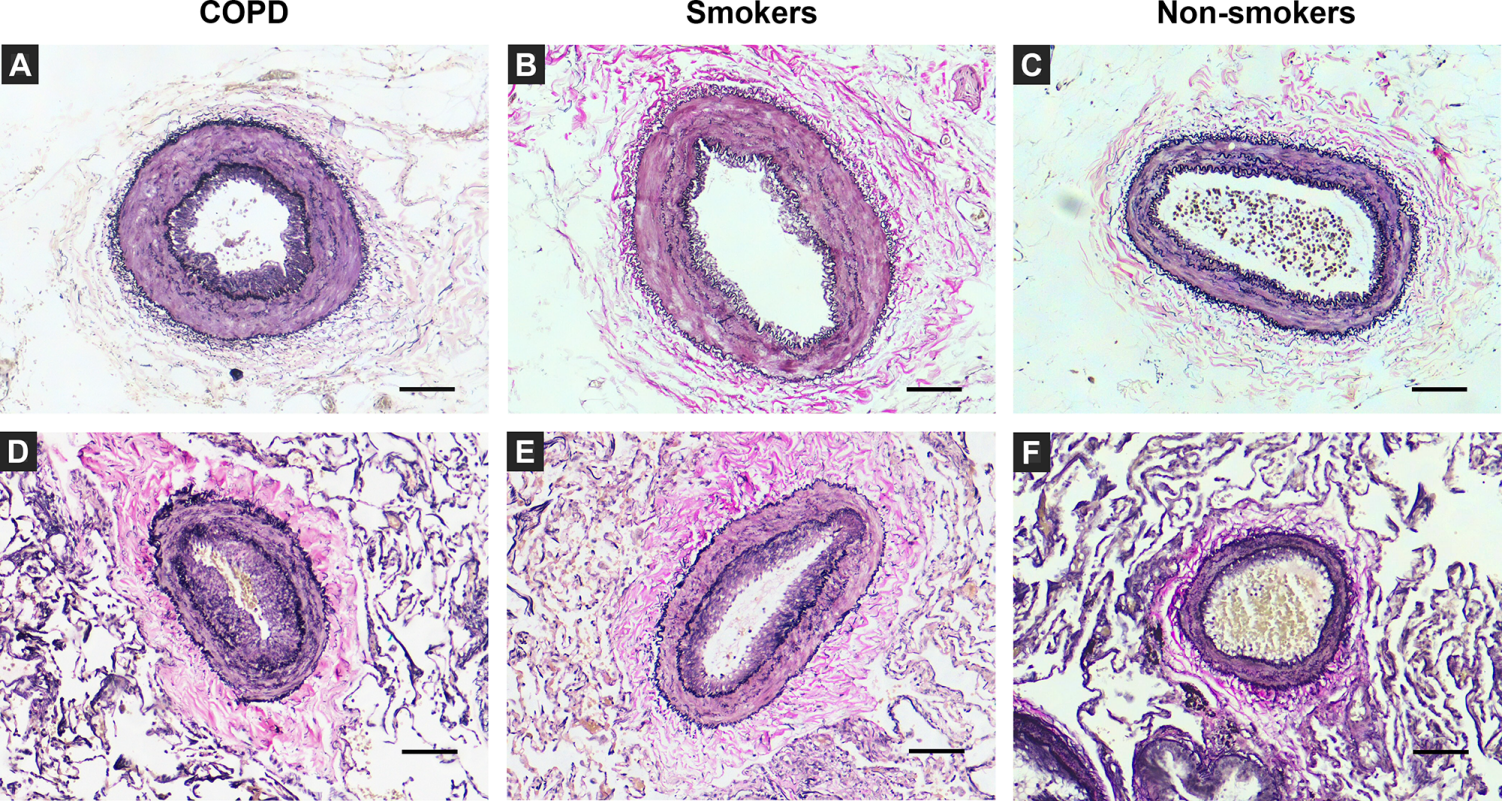
Representative photomicrographs of orcein stained elastin fiber in cross sections of intercostal and muscular pulmonary arteries. Images from A to C represent intercostal arteries sections. The intima in **A** (COPD) is thicker than in the smokers (**B**) and the non-smokers (**C**) group. Images from D to F show the muscular pulmonary arteries sections of COPD (**D**), smokers (**E**), and non-smokers (**F**). Vessel remodelling, characterized by thickening of the intima layer, is more evident in the COPD group. Elastin-orcein stain, scale bar = 100μm.

Significant correlations were observed in the %IA between systemic and pulmonary arteries (Spearman’s rho = 0.435, p = 0.016) in the overall population. However no correlation was observed between systemic and pulmonary arteries in %LN (Spearman’s rho = 0.360, p = 0.051), ITI (Spearman’s rho = 0.145, p = 0.445) and IMR (Spearman’s rho = 0.061, p = 0.749). In Fig 3, microphotographs of systemic and muscular pulmonary arteries with ascending intimal thickening, belonging to the three groups of study are represented.

## Discussion

The present investigation is, to the best of our knowledge, the first histo-morphometric analysis performed with the aim of evaluating concomitantly the intimal thickening of the systemic and pulmonary arteries of COPD subjects compared to smokers. The principal findings of this study can be summarized as follows: 1) Patients with COPD have an increased %IA of systemic and pulmonary arteries compared to smokers with normal lung function; 2) %IA of systemic arteries is independently related to gender, the presence of metabolic syndrome and COPD, while the %IA of pulmonary arteries is related only with COPD; 3) there is a correlation between the %IA of systemic and pulmonary arteries in the overall population.

Previous studies have shown that the intimal thickening of systemic arteries is due to the increased formation of extracellular matrix (ECM), which includes an increased secretion and deposition of proteins, growth factors and enzymes that regulate ECM.[14] These remodeling changes could contribute to different clinical vascular disorders such as multifactorial atherosclerosis.[15] In the context of the current study, four methods were used to evaluate the propensity of in situ IC artery and muscular pulmonary arteries to develop intimal thickening: %IA, %LN, ITI and IMR. The %IA, %LN and ITI indices are measures of the severity of intimal thickening that allow for an accurate evaluation of eccentric or irregular disease since they consider areas rather than widths. Also, they could be useful for comparing intimal thickening between different vascular beds.[12] IMR is an alternative method for comparing intimal disease, and it specifically evaluates the severity of atherosclerosis, in which the maximal thickness of intima from cross sectional is considered and is presumed to be a useful parameter for comparisons of the same artery among different patients but cannot be used to compare different vascular beds.[12] In the present study, it was found that COPD subjects exhibit an increased intimal thickening of systemic arteries in terms of %IA and a numerically increasing trend in terms of %LN and ITI compared to smokers; all three of these parameters are area-dependent. Nonetheless, no differences were found in IMR, which could be explained by the irregular intimal thickening observed. Overall, these results support the idea that COPD patients exhibit a major intimal hyperplasia in systemic circulation compared to smokers with normal lung function. This novel data is of particular relevance since these vascular changes could be the initial lesions in a complex process resulting in atherosclerosis or the beginning of a non-atherosclerosis related subclinical vascular disease.[16] In line with this, prior studies using different non-invasive measurements to evaluate subclinical vascular disease determined an increased cardiovascular risk in stable COPD subjects independent of age or smoking status.[17–20] Specifically, the ultrasonographic evaluation of vascular remodelling showed that carotid artery wall thickening (carotid intima-media thickness) was associated with the severity of airflow limitation in COPD subjects.[17–20] Similarly, there are data supporting the association of more advanced vascular disease in elderly subjects with COPD, assessed by high-resolution magnetic resonance imaging, where COPD was an independent predictor for the presence of a lipid core, and therefore of vulnerable plaque in the carotid artery.[17] Of note, the current analysis of IC arteries of the non-smoker and smoker group show a similar intimal layer as in morphometric studies in post-mortem subjects who had died of non-cardiac diseases,[21] which gives consistence to our results.

Most of the studies focussing on atherosclerosis have been performed in arterial tissue from arterectomies or are post-mortem studies carried out in advanced stages of the disease.[21] However, a valid alternative to studying the initial processes is the evaluation of the 5^th^ IC artery which arises from the descending thoracic aorta and provides blood supply to the ribs, IC spaces and skin of the anterolateral thoracic wall.[22] Of all IC arteries, the 5^th^ IC artery has the greatest luminal diameter, length and rate of flow,[23] which makes it accessible in all patients undergoing open thoracotomy. Also, of the three segments of the IC artery: proximal, middle and distal, the middle level (analyzed in this study), has a thin inner layer and a muscular or elasto-muscular media layer, characterized by an absence or by few elastic laminas except for the IEL and EEL, respectively.[21] These two features result in a greater tendency toward intimal hyperplasia as compared to elastic conduits,[13,15] therefore muscular IC artery appears histologically more in the coronary arteries or radial arteries, where remodelling is most evident.[15,21,23]

Risk factors for the intimal thickening of systemic arteries vary widely between morphometric studies; in general the clinical variables tested are the same ones that are related to clinical atherosclerosis. However, previous studies have demonstrated that different arterial beds have different risk factors for the development of intimal hyperplasia and atherosclerosis.[13] The most common clinical factors associated with intimal hyperplasia in different arteries (non IC artery), as measured by different methods are: diabetes mellitus, renal failure, hypertension, peripheral vascular disease, smoking and age.[13] In our study, the factors associated with the intimal thickening of the IC artery as measured by %IA were female gender, metabolic syndrome and COPD status. Although female gender may have different features in the functional and morphometric behavior of systemic arteries, as previously reported;[24] in our study, the poor representation of women in some of the groups analyzed makes the interpretation of results difficult, as we discussed in the limitations section. In pulmonary circulation, the results of an increased intimal thickening of the muscular pulmonary arteries of COPD subjects are in agreement with prior morphometric studies that have demonstrated a higher proliferation of smooth muscles cells and the deposition of ECM proteins in the intima layer of the pulmonary vessels in COPD subjects.[7,25]

Another novel issue in this study is the relationship between systemic and pulmonary vascular remodeling changes assessed by histo-morphometric analysis. Previous studies evaluating pulmonary vascular disease (measured by the cross sectional area of small pulmonary vessels in computerized tomography) or histological pulmonary atherosclerosis (in post-mortem analysis) showed a positive correlation with the atherosclerosis of systemic arteries.[26–27] Although the underlying mechanisms are still unknown, it is hypothesized, that systemic inflammation and endothelial dysfunction may promote both systemic and pulmonary vascular alterations, and may lie behind the close relationship between both conditions.[28] As previously reported in systemic sclerosis, an immunologic disorder with a simultaneous impairment of systemic and pulmonary circulation could occur.[29]

### Limitations

The principal limitation of the study is the poor representation of female gender in the COPD and smokers groups due to the baseline characteristics of our population (patients with lung carcinoma and a major smoking habit are mostly male patients). Therefore, this gender misbalance makes it difficult to draw conclusions about gender beyond spurious associations. The population of the study has primary, treatable lung cancer; therefore lung cancer could be a possible introduced bias. However, we are assuming any effect would be the same across all the subjects included in the study. It is important to consider that it would be impossible to obtain lung tissue and arterial samples from living patients, if it were not for the indication of the surgery. Another possible limitation of the study is that, due to its observational design, causal conclusions or strong conclusions cannot be drawn beyond observing a significant association between the presence of COPD and vascular remodelling changes in systemic and pulmonary arteries.

### Conclusions

In conclusion, the current study shows a greater intimal thickening in systemic and pulmonary arteries in the COPD group compared to smokers group. These histopathological observations imply that subjects with a mild-moderate COPD in addition to pulmonary vascular involvement, also show systemic vascular changes (histological remodelling) which could explain the high prevalence of cardiovascular disease in COPD patients. Therefore, in clinical practice, the adequate management of pulmonary disease and others modifiable cardiovascular risk factors should be a target in all subjects with COPD, even in mild stages, in order to minimize cardiovascular consequences. The present study provides a model for future studies involving initial remodelling changes in both systemic and pulmonary circulations.

## Supporting Information

S1 File Additional definitions and details of analyses. (DOCX)

## References

[1] Vestbo J, Hurd SS, Agustí AG, Jones PW, Vogelmeier C, Anzueto A, et al Global strategy for the diagnosis, management, and prevention of chronic obstructive pulmonary disease: GOLD executive summary. Am J Respir Crit Care Med. 2013;187: 347–365. 10.1164/rccm.201204-0596PP 22878278

[2] Soriano JB, Visick GT, Muellerova H, Payvandi N, Hansell AL. Patterns of comorbidities in newly diagnosed COPD and asthma in primary care. Chest. 2005;128: 2099–2107. 16236861 10.1378/chest.128.4.2099

[3] Van Eeden S, Leipsic J, Paul Man SF, Sin DD. The relationship between lung inflammation and cardiovascular disease. Am J Respir Crit Care Med. 2012;186: 11–16. 10.1164/rccm.201203-0455PP 22538803

[4] Sin DD, Man SF. Chronic obstructive pulmonary disease: a novel risk factor for cardiovascular disease. Can J Physiol Pharmacol. 2005;83: 8–13. 15759045 10.1139/y04-116

[5] Hole DJ, Watt GC, Davey-Smith G, Hart CL, Gillis CR, Hawthorne VM. Impaired lung function and mortality risk in men and women: findings from the Renfrew and Paisley prospective population study. BMJ. 1996;313: 711–716. 8819439 10.1136/bmj.313.7059.711 PMC2352103

[6] Magee F, Wright JL, Wiggs BR, Paré PD, Hogg JC. Pulmonary vascular structure and function in chronic obstructive pulmonary disease. Thorax. 1988 3;43: 183–189. 3406902 10.1136/thx.43.3.183 PMC461159

[7] Santos S, Peinado VI, Ramírez J, Melgosa T, Roca J, Rodriguez-Roisin R, et al Characterization of pulmonary vascular remodelling in smokers and patients with mild COPD. Eur Respir J. 2002;19: 632–638. 11998991 10.1183/09031936.02.00245902

[8] Barberà JA, Peinado VI, Santos S. Pulmonary hypertension in chronic obstructive pulmonary disease. Eur Respir J. 2003;21: 892–905. 12765440 10.1183/09031936.03.00115402

[9] Hsia CC, Hyde DM, Ochs M, Weibel ER; ATS/ERS Joint Task Force on Quantitative Assessment of Lung Structure. An official research policy statement of the American Thoracic Society/European Respiratory Society: standards for quantitative assessment of lung structure. Am J Respir Crit Care Med. 2010;181: 394–418. 10.1164/rccm.200809-1522ST 20130146 PMC5455840

[10] Alberti KG, Eckel RH, Grundy SM, Zimmet PZ, Cleeman JI, Donato KA, et al Harmonizing the metabolic syndrome: a joint interim statement of the International Diabetes Federation Task Force on Epidemiology and Prevention; National Heart, Lung, and Blood Institute; American Heart Association; World Heart Federation; International Atherosclerosis Society; and International Association for the Study of Obesity. Circulation. 2009;120: 1640–1645. 10.1161/CIRCULATIONAHA.109.192644 19805654

[11] Cook TA, Yates PO. A critical survey of techniques for arterial mensuration. J Pathol. 1972;108: 119–127. 4647504 10.1002/path.1711080205

[12] Chowdhury UK, Airan B, Mishra PK, Kothari SS, Subramaniam GK, Ray R, et al Histopathology and morphometry of radial artery conduits: basic study and clinical application. Ann Thorac Surg. 2004;78: 1614–1621. 15511443 10.1016/j.athoracsur.2004.03.105

[13] Ruengsakulrach P, Sinclair R, Komeda M, Raman J, Gordon I, Buxton B. Comparative histopathology of radial artery versus internal thoracic artery and risk factors for development of intimal hyperplasia and atherosclerosis. Circulation. 1999;100 (19 Suppl):II139–144. 10567293 10.1161/01.cir.100.suppl_2.ii-139

[14] Subbotin VM. Analysis of arterial intimal hyperplasia: review and hypothesis. Theor Biol Med Model. 2007;4:41 17974015 10.1186/1742-4682-4-41 PMC2169223

[15] Barry M, Touati G, Chardon K, Laude M, Libert JP, Sevestre H. Histologic study of coronary, radial, ulnar, epigastric and internal thoracic arteries: application to coronary artery bypass grafts. Surg Radiol Anat. 2007;29: 297–302. 17505775 10.1007/s00276-007-0214-4

[16] Cizek SM, Bedri S, Talusan P, Silva N, Lee H, Stone JR. Risk factors for atherosclerosis and the development of preatherosclerotic intimal hyperplasia. Cardiovasc Pathol. 2007;16: 344–350. 18005873 10.1016/j.carpath.2007.05.007 PMC2185541

[17] Lahousse L, van den Bouwhuijsen QJ, Loth DW, Joos GF, Hofman A, Witteman JC, et al Chronic obstructive pulmonary disease and lipid core carotid artery plaques in the elderly: the Rotterdam Study. Am J Respir Crit Care Med. 2013;187: 58–64. 10.1164/rccm.201206-1046OC 23144329

[18] Chindhi S, Thakur S, Sarkar M, Negi PC. Subclinical atherosclerotic vascular disease in chronic obstructive pulmonary disease: Prospective hospital-based case control study. Lung India. 2015;32: 137–141. 10.4103/0970-2113.152624 25814798 PMC4372867

[19] van Gestel YR, Flu WJ, van Kuijk JP, Hoeks SE, Bax JJ, Sin DD, et al Association of COPD with carotid wall intima-media thickness in vascular surgery patients. Respir Med. 2010;104: 712–716. 10.1016/j.rmed.2009.10.027 19942421

[20] Iwamoto H, Yokoyama A, Kitahara Y, Ishikawa N, Haruta Y, Yamane K, et al Airflow limitation in smokers is associated with subclinical atherosclerosis. Am J Respir Crit Care Med. 2009;179: 35–40. 10.1164/rccm.200804-560OC 18931335

[21] van Son JA, Smedts F, Korving J, Guyt A, de Kok LB. Intercostal artery: histomorphometric study to assess its suitability as a coronary bypass graft. Ann Thorac Surg. 1993;56: 1078–1081. 8239803 10.1016/0003-4975(95)90018-7

[22] Arnold M. The surgical anatomy of sternal blood supply. J Thorac Cardiovasc Surg. 1972;64: 596–610. 4562531

[23] John LC, Chan CL, Anderson DR. Potential use of the intercostal artery as an in situ graft: a cadaveric study. Ann Thorac Surg. 1995;59: 190–195. 7818322 10.1016/0003-4975(94)00715-J

[24] Mong K, Duggan JA, Tabrizchi R. Comparative study of functional responses and morphometric state of distal radial arteries in male and female. Ann Thorac Surg. 2002;74: 2126–2131. 12643406 10.1016/s0003-4975(02)03984-x

[25] Wright JL, Petty T, Thurlbeck WM. Analysis of the structure of the muscular pulmonary arteries in patients with pulmonary hypertension and COPD: National Institutes of Health nocturnal oxygen therapy trial. Lung. 1992;170: 109–124. 1501507 10.1007/BF00175982

[26] Matsuoka S, Yamashiro T, Diaz A, Estépar RS, Ross JC, Silverman EK, et al The relationship between small pulmonary vascular alteration and aortic atherosclerosis in chronic obstructive pulmonary disease: quantitative CT analysis. Acad Radiol. 2011;18: 40–46. 10.1016/j.acra.2010.08.013 20947389 PMC3006041

[27] Moore GW, Smith RR, Hutchins GM. Pulmonary artery atherosclerosis: correlation with systemic atherosclerosis and hypertensive pulmonary vascular disease. Arch Pathol Lab Med. 1982;106: 378–380. 6213213

[28] Vukic Dugac A, Ruzic A, Samarzija M, Badovinac S, Kehler T, Jakopovic M. Persistent endothelial dysfunction turns the frequent exacerbator COPD from respiratory disorder into a progressive pulmonary and systemic vascular disease. Med Hypotheses. 2015;84: 155–158. 10.1016/j.mehy.2014.11.017 25539899

[29] Irzyk K, Bienias P, Rymarczyk Z, Bartoszewicz Z, Siwicka M, Bielecki M, et al Assessment of systemic and pulmonary arterial remodelling in women with systemic sclerosis. Scand J Rheumatol. 2015;44: 385–388. 10.3109/03009742.2015.1021710 25928303

