# Supplementary material for: Systemic and Pulmonary Vascular Remodelling in Chronic Obstructive Pulmonary Disease
Source: PLoS One. 2016 Apr 5;11(4):e0152987. doi: 10.1371/journal.pone.0152987 (PMC4821623; doi:10.1371/journal.pone.0152987)
Supplement: S1 File — (DOCX) [file pone.0152987.s001.docx]

**Supporting Information**

**S1 File. Additional definitions and details of analyses.**

**Metabolic Syndrome**

Metabolic syndrome (MetS) was defined as the presence of three or more of the metabolic parameters in accordance with current criteria.[9] The metabolic parameters were: 1) waist circumference (≥88 cm in females, ≥102 cm in males); 2) systolic blood pressure ≥130 and/or diastolic blood pressure ≥85 mmHg or antihypertensive treatment; 3) FBG ≥5.6 mmol/L^−1^ or anti-diabetic treatment; 4) triglycerides ≥1.7 mmol/L^−1^ or lipid-lowering treatment; 5) cHDL <1.3 mmol/L^−1^ in males and <1 mmol/L^−1^ in females or lipid-lowering treatment.

**Index of arterial narrowing**

Index of narrowing was estimated as the ratio between the measured total area and that extrapolated from the theoretical distended diameter: [*theoretical diameter = length of the external elastic lamina / pi (π)*].[10]

**Linear regression analyses**

Linear regression analyses following the backward method were performed to determine which clinical factors were associated with intimal thickening, where the dependent variable was %IA. The variables included in the regression model of systemic intimal thickening were : male gender, age, body mass index (BMI), pack-years, emphysema, systemic hypertension, diabetes mellitus, metabolic syndrome (MetS) , lipid lowering therapy, angiotensin-converting enzyme inhibitors or angiotensin II receptor blockers (ACEIs/ARBs) therapy, high-density lipoprotein cholesterol (cHDL), low-density lipoprotein cholesterol (cLDL), triglycerides, leukocytes and COPD. The variables included in the regression model of pulmonary intimal thickening were: male gender, age, BMI, pack-years, emphysema, systemic hypertension, diabetes mellitus, MetS, lipid lowering therapy, ACEIs/ARBs, inhaled corticosteroids, leukocytes and COPD. The resulting factors were considered as covariables in the ANCOVA method used to evaluate the primary endpoint and all other between-groups comparisons.
